# Cardiovascular Magnetic Resonance Parametric Mapping Techniques for the Assessment of Chronic Coronary Syndromes

**DOI:** 10.3390/jcdd9120443

**Published:** 2022-12-09

**Authors:** Maria Anna Bazmpani, Chrysovalantou Nikolaidou, Christos A. Papanastasiou, Antonios Ziakas, Theodoros D. Karamitsos

**Affiliations:** 1 Department of First Cardiology, Aristotle University of Thessaloniki School of Medicine, AHEPA University Hospital, 54636 Thessaloniki, Greece; 2 Oxford Centre for Clinical Magnetic Resonance Research, University of Oxford, Oxford OX3 9DU, UK

**Keywords:** chronic coronary syndromes, stress T1 mapping, quantitative stress CMR, myocardial blood flow, myocardial perfusion reserve

## Abstract

The term chronic coronary syndromes encompasses a variety of clinical presentations of coronary artery disease (CAD), ranging from stable angina due to epicardial coronary artery disease to microvascular coronary dysfunction. Cardiac magnetic resonance (CMR) imaging has an established role in the diagnosis, prognostication and treatment planning of patients with CAD. Recent advances in parametric mapping CMR techniques have added value in the assessment of patients with chronic coronary syndromes, even without the need for gadolinium contrast administration. Furthermore, quantitative perfusion CMR techniques have enabled the non-invasive assessment of myocardial blood flow and myocardial perfusion reserve and can reliably identify multivessel coronary artery disease and microvascular dysfunction. This review summarizes the clinical applications and the prognostic value of the novel CMR parametric mapping techniques in the setting of chronic coronary syndromes and discusses their strengths, pitfalls and future directions.

## 1. Introduction

Chronic coronary syndromes is a term which encompasses various clinical presentations of coronary artery disease (CAD), including patients with established or suspected CAD and stable anginal symptoms, patients with angina due to microvascular dysfunction or coronary vasospasm, asymptomatic patients after initial diagnosis and treatment and patients with left ventricular systolic dysfunction [1]. According to the current European and American guidelines on chronic coronary syndromes [1,2], the selection of appropriate testing should be guided by the clinical likelihood of CAD. Patients with an intermediate pre-test probability or with confirmed CAD should undergo non-invasive functional testing for ischemia with a high accuracy for detecting CAD, such as stress echocardiography, single photon emission tomography (SPECT), cardiovascular magnetic resonance (CMR) or positron emission tomography (PET) [3].

Stress CMR can detect differences in myocardial perfusion with a high degree of precision by utilizing first-pass imaging of gadolinium-based contrast agents during pharmacological stress [4,5,6,7]. Moreover, CMR T1 mapping techniques at rest and after vasodilator stress offer the unique capability of tissue characterization and ischemia testing without the need for gadolinium contrast administration. Each pixel of the generated maps represents an absolute T1 value on a pixel-by-pixel basis. The resulting color-coded maps facilitate the visual differentiation of normal from diseased myocardium [8,9,10,11]. Furthermore, novel automated perfusion mapping techniques allow quantification of myocardial blood flow (MBF) and myocardial perfusion reserve (MPR) and facilitate the diagnosis of epicardial CAD as well as of microvascular dysfunction [12,13,14,15], the detection of which is often challenging with other non-invasive imaging modalities.

Taking into consideration the recent advances in parametric mapping and quantitative perfusion CMR techniques, the aim of the current review is to summarize their clinical applications in the detection of chronic coronary syndromes, highlighting their strengths and pitfalls, and to explore their potential future role as the imaging modality of choice for the assessment of stress-induced myocardial ischemia.

## 2. T1 Mapping

### 2.1. Principles of T1 Mapping

T1 allows measurement of the longitudinal or spin-lattice relaxation time, which reflects the mobility of protons and the time required to recover to their thermal equilibrium after being excited by magnetization [16]. With the application of modern sequences, T1 values measured in vivo are displayed pixel-wise as T1 maps which allow quantitative tissue characterization and visual interpretation [17]. T1 measurements reflect intrinsic tissue properties with each tissue type exhibiting a normal range of T1 values. Therefore, focal or diffusely diseased myocardium due to changes either in molecular environment (fibrosis, amyloid, iron or lipid deposition) [10,18,19] or excessive water content (edema) [20,21] can be differentiated from the normal myocardium.

Myocardial T1 values are affected by technical parameters, including partial volume effects and magnetic field strength, as well as physiologic parameters such as age, gender and heart rate [16,22]. T1 measurements are performed using the modified look-locker inversion recovery (MOLLI) sequence which, in contrast to the look-locker approach, enables selective data acquisition and merging of image sets from multiple look-locker experiments in one data set [17,23]. Shortened MOLLI (ShMOLLI) generates myocardial T1 maps in a short breath-hold [22,24] and less heart rate dependency for long T1 compared to MOLLI [25,26]. Most recently, the saturation recovery single-shot acquisition (SASHA) sequence has been validated for in vivo T1 mapping, allowing fast measurements independently of heart rate and flip angle [27]. Free-breathing SASHA T1 mapping has also shown excellent results for acquisitions longer than 30 s [28]. Another hybrid pulse sequence, referred to as saturation pulse prepared heart-rate-independent inversion recovery (SAPPHIRE), has shown superior results in yielding accurate T1 maps that require shorter breath holds compared to MOLLI, with promising future applications [29].

### 2.2. Vasodilator Pharmacological Stressors for Stress CMR

Stress CMR is usually performed using three vasodilatory agents: adenosine, dipyridamole, and regadenoson. Adenosine, a non-selective adenosine receptor agonist with a quick onset of action, is administered as a continuous infusion at doses ranging from 140 mcg/kg/min to 210 mcg/kg/min in order to achieve the desired hemodynamic response [30]. Adenosine is safe and generally well-tolerated but is contraindicated in patients with uncontrolled asthma or severe chronic obstructive pulmonary disease, second- or third-degree atrioventricular block, hypotension (systolic blood pressure < 90 mmHg), recent acute coronary syndrome, severe bilateral carotid stenosis and decompensated heart failure [30,31]. Dipyridamole, which works by blocking the metabolism of endogenous adenosine, can also be used for vasodilator stress perfusion, with similar contraindications to adenosine. Regadenoson is a novel selective low-affinity A_2a_-specific receptor agonist which is increasingly used due to its safety profile and ease of administration [32]. It is administered as a slow bolus injection of 400 mcg, irrespective of body weight. Most common adverse reactions include dyspnea, headache, flushing, gastrointestinal symptoms and conduction abnormalities, and they usually resolve within 30 min. Regadenoson is safer than adenosine for patients with asthma or chronic obstructive pulmonary disease (COPD) [33]. However, contrary to adenosine, evaluation of positive response with the splenic switch off sign is not possible [34]. The above agents act as coronary vasodilators and facilitate the detection of inducible myocardial perfusion defects in areas supplied by stenotic coronary artery branches [35]. Withdrawal from caffeine for 24 h is required, as caffeine inhibits their vasodilatory action [36].

### 2.3. Clinical Applications of Stress T1 Mapping in Chronic Coronary Syndromes

Native stress T1 mapping has emerged as a useful diagnostic tool for the detection of CAD. Myocardial blood volume (MBV) represents myocardial water content both in macro- and microcirculation. It constitutes approximately 10% of the total myocardial volume at rest and may increase two-fold during coronary vasodilatory stress [37]. Compared to myocardial blood flow (MBF), MBV provides further information on significant coronary artery stenosis and myocardial viability showing a better association with myocardial oxygen demand [38]. Based on the notion that increased MBV is expected to increase T1 values, a proof-of-concept study demonstrated that adenosine rest and stress T1 mapping allows differentiation of normal, infarcted, ischemic and remote myocardium without the need for gadolinium contrast administration [39]. The difference in T1 values during stress and rest (δΤ1) was defined as T1 reactivity and showed four distinctive patterns. In healthy volunteers, increased myocardial T1 values were noted during vasodilatory stress. On the other hand, no significant stress δΤ1 reactivity was observed for infarcted and ischemic myocardium. Interestingly, remote myocardium in patients with ischemia showed a blunted stress T1 response, possibly reflecting microvascular dysfunction. This was further investigated by Levelt et al., who demonstrated that in well-controlled diabetic patients without obstructive CAD, there was a blunted T1 response during the adenosine stress test that was attributed to microvascular abnormalities [15]. Regadenoson stress and rest T1 mapping was demonstrated to be a viable alternative to adenosine in the distinction of normal, ischemic, infarcted and remote myocardium [40].

Bohnen et al. conducted a study on the performance of non-contrast stress T1 mapping in detecting myocardial ischemia in 100 patients with suspected or known CAD. Myocardium with inducible ischemia demonstrated no significant T1 reactivity, suggesting that MBV is maximally increased at rest in areas where there is severe coronary artery stenosis. Focal regions from areas of interest were more sensitive in depicting ischemia compared to the American Heart Association segmental model [41]. Van Assen et al. showed that T1 reactivity was significantly different between normal and ischemic or infarcted myocardium; however, there was not a statistically significant difference between infarcted and ischemic myocardium. Nonetheless, the investigators suggested that infarcted myocardium may be differentiated from ischemic by taking into consideration the much higher native T1 values of infarcted myocardium at rest [42]. A study by Yimcharoen et al. further validated the above findings in a larger cohort of 181 participants that underwent rest and stress T1 mapping using MOLLI. An increase in T1 values under stress was demonstrated for normal subjects, whereas patients with ischemic and infarcted myocardium had elevated T1 values at rest without any significant T1 reactivity with stress. Differentiation of infarcted myocardium based on resting T1 and of ischemic myocardium based on T1 reactivity during stress showed high diagnostic accuracy [43]. Finally, Gezmis et al. demonstrated that MOLLI and ShMOLLI sequences do not differ significantly regarding T1 reactivity of remote, ischemic and infarcted myocardium.

### 2.4. Chronic Myocardial Infarction Imaging with T1 Mapping

Pre- and post-contrast T1 mapping have been shown to have clinical utility in chronic myocardial infarction. For example, Messroghli et al. demonstrated that pre-contrast T1 values of infarcted myocardium were higher than the ones of remote areas but not as high as in the setting of acute myocardial infarction. Post-contrast T1 mapping also showed high sensitivity and specificity in discriminating infarcted segments as the hyperenhanced areas demonstrated shorter T1 times compared to remote areas after administration of gadolinium contrast agent and allowed for accurate measurement of the infract size [23]. Additionally, T1 mapping without the use of contrast agent can detect fatty infiltration of an infarcted area by demonstrating low T1 values within this area. Virtual native enhancement (VNE) is a novel artificial intelligence technique which produces LGE-like images without contrast administration. Zhang et al. demonstrated VNE provided high agreement with LGE images for myocardial scar assessment in patients with previous myocardial infarction with superior image quality [44].

### 2.5. Prognostic Role of T1 Mapping in CAD

The prognostic role of T1 mapping in CAD remains unclear. Puntmann et al. demonstrated that native T1 of non-infarcted myocardium was significantly elevated in patients with CAD and was the sole independent predictor of all-cause mortality, superior to commonly used biomarkers, including myocardial volumes, left ventricular ejection fraction and clinical scores [45].The importance of this study lies in the fact that what was previously considered as “normal” myocardium seems to undergo pathophysiological changes associated with prognosis and may serve as a future therapeutic target.

### 2.6. Advantages and Limitations of T1 Mapping and Stress T1 Mapping

The main advantage of T1 mapping is that it eliminates the need for gadolinium contrast, which is contraindicated in people with known allergy to gadolinium and should be avoided in certain patient groups, such as those with severe renal impairment or pregnant/breast-feeding women. Rest and stress T1 mapping offer the unique potential to assess myocardial ischemia, the coronary vasodilatory reserve, and the health of the coronary microcirculation with a single, non-invasive test and without exposure to ionizing radiation.

Limitations of T1 mapping arise from the several factors that affect the accuracy of T1 measurements. Protocol parameters, sequence design, field strength and scanner adjustments, fit model, tissue characteristics and patient characteristics may lead to variations in normal values and influence reproducibility [46]. MOLLI and ShMOLLI are most commonly used and have demonstrated higher precision for T1 mapping than SASHA and SAPPHIRE, which may be further optimized in the future [47]. Heart rate variations, which are of particular importance in stress T1 mapping, is another factor that potentially introduces errors in T1 measurements and T1 reactivity. SASHA protocol is heart rate independent, but it has not been extensively studied in the field of rest/stress T1 mapping. Magnetization transfer is another factor which affects the accuracy of inversion recovery methods as well as motion artefacts that may result from longer breath-holds or periods of motion during stress [48]. Finally, selection and drawing the region of interest (ROI) may result in variations in measured T1 values.

## 3. Quantitative Myocardial Perfusion Mapping

### 3.1. Newer Automated Techniques for CMR Perfusion Quantification

**Figure 1:**
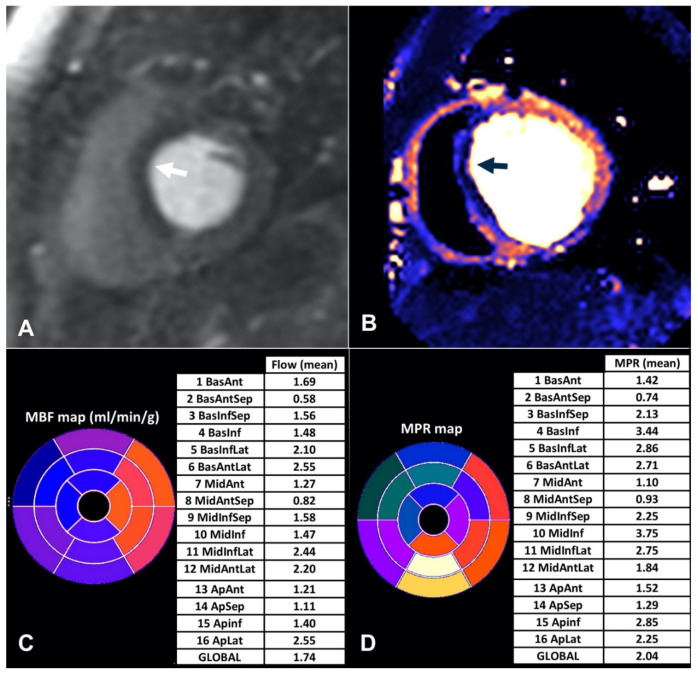
Visual and quantitative analysis of stress myocardial perfusion in a patient with hypoperfusion in the septum. (**A**) First-pass perfusion images showing a hypointense area in the basal septum (arrow). (**B**) Pixel-wise map of myocardial blood flow map demonstrating the region of low flow in darker color than normal flow (arrow). (**C**,**D**) Polar maps of myocardial blood flow (MBF) and myocardial perfusion reserve (MPR) demonstrating low values in the left anterior descending coronary artery territory.

Qualitative stress perfusion CMR has demonstrated a high diagnostic accuracy in the detection of significant CAD [49,50], with an incremental prognostic value on top of clinical risk factors and wall motion abnormalities [51,52,53]. Nonetheless, it has some inherent limitations as it is operator-dependent and may miss the diagnosis of multivessel disease. Semi-quantitative methods for myocardial perfusion have been developed but yielded almost similar diagnostic accuracy to qualitative approaches at the expense of the time required for analysis. Furthermore, they may underestimate perfusion at higher flow rates [54]. Thus, their implementation in clinical practice remained limited. The recent development of quantitative myocardial perfusion CMR provided the solution to the problems of qualitative and semi-quantitative methods. In its early days, quantitative myocardial perfusion mapping posed many challenges. Accurate measurement of arterial input function and conversion of signals into contrast concentrations was time consuming and rendered repeatability cumbersome [55,56]. Another pitfall was the lack of linearity between contrast concentration and intensity of signal, especially at higher concentrations of contrast. A fully automated quantitative technique which overcame these obstacles and generates first-pass perfusion images and a pixel-wise map of myocardial blood flow in only a few minutes was developed by Kellman et al. [57] (Figure 1). The myocardial perfusion reserve (MPR) and MBF measurements with the automated perfusion mapping CMR were validated in healthy subjects and demonstrated similar repeatability to PET [13].

### 3.2. Clinical Applications of Quantitative Stress CMR—What We Know So Far

#### 3.2.1. Validation and Diagnostic Accuracy

Several studies have shown that quantitative stress perfusion CMR can reliably identify significant CAD with a similar diagnostic accuracy to conventional methods. Lockie et al. compared MPR calculated by quantitative analysis of perfusion CMR data performed at 3T with invasively measured fractional flow reserve and demonstrated that an MPR of 1.58 provided a sensitivity of 0.80 and specificity of 0.89 in discriminating functionally significant from non-significant CAD [58]. Mordini et al. compared fully quantitative perfusion CMR, semi-quantitative and qualitative perfusion CMR methods against quantitative coronary angiography. Fully quantitative stress perfusion CMR yielded a high diagnostic accuracy for the detection of obstructive CAD with a sensitivity of 87% and specificity of 93%, significantly outperforming semi-quantitative and qualitative measures [59]. Morton et al. compared MPR measured on CMR to MPR measured by PET and found a strong correlation between the two methods [60]. Similarly, Engblom et al. showed that there is a strong correlation and good agreement between CMR and PET with regards to regional and global myocardial perfusion and MPR [61]. A later study by Hsu et al. showed that quantification of MBF and MPR by a fully automated pixel-wise quantitative perfusion CMR sequence had excellent diagnostic accuracy on a per-patient basis and very good diagnostic accuracy on a per-vessel basis when compared to quantitative coronary angiography. Recently, a single-center, prospective study demonstrated that a novel technique of quantitative simultaneous multislice stress myocardial perfusion with iterative reconstruction in patients with suspected CAD showed a high diagnostic accuracy for the detection of ischemia [62].

On the contrary, two sub-studies of the CE-MARC trial in a small number of patients showed that while quantitative perfusion CMR indeed has a high diagnostic accuracy for CAD, its diagnostic value may not necessarily outweigh qualitative visual analysis [63,64].

#### 3.2.2. Three-Vessel CAD

Three-vessel CAD is characterized by balanced ischemia which can be difficult to detect with functional imaging, especially with SPECT myocardial perfusion imaging [65]. Recent data suggest that automated pixel-wise quantitative CMR perfusion mapping has good diagnostic accuracy in detecting multivessel CAD as well as microvascular dysfunction [12]. Kotecha et al. found that in patients with two-vessel and three-vessel disease, perfusion CMR mapping was superior to qualitative visual assessment in correct identification of perfusion defects. More specifically, in patients with visual perfusion defects on stress perfusion CMR but no regional hypoperfusion, a global stress MBF < 2.25 mL/g/min can reliably detect obstructive three-vessel disease. On the other hand, an MBF < 1.94 mL/g/min matching coronary artery distribution in patients with regional perfusion defects is suggestive of obstructive single- or two-vessel disease [66]. Further multicenter clinical trials are warranted to explore the role of quantitative perfusion CMR mapping in multivessel disease and in guiding revascularization management among these patients.

#### 3.2.3. Microvascular Dysfunction

**Figure 2:**
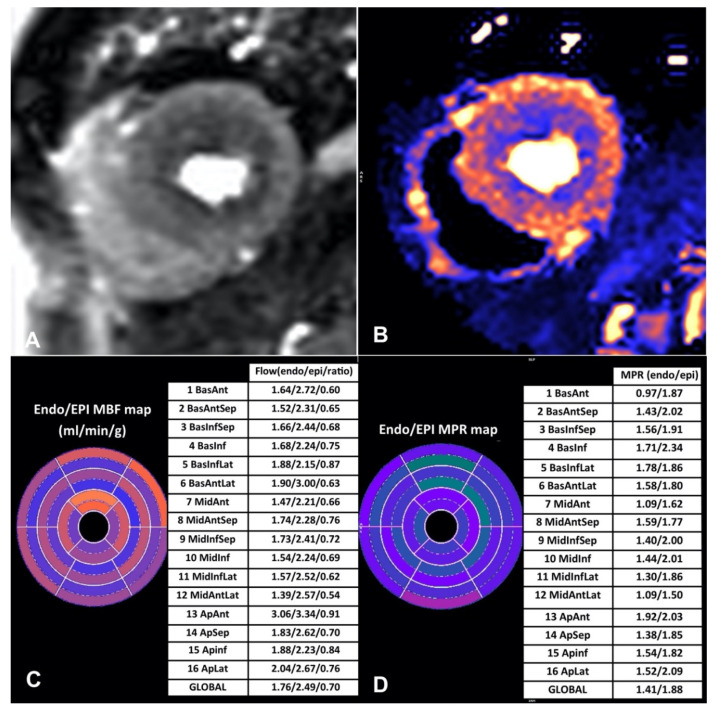
Visual and quantitative stress myocardial perfusion analysis in a patient with microvascular dysfunction. (**A**) First-pass perfusion images showing a circumferential hypointense area in the endocardium. (**B**) Pixel-wise map of myocardial blood flow map demonstrating the regions of low flow in darker color than normal flow. (**C**,**D**) Polar maps of myocardial blood flow (MBF) and myocardial perfusion reserve (MPR) demonstrating low values in the endocardium compared to normal values in the epicardial segments.

Microvascular dysfunction is characterized by impaired autoregulation of arterial microcirculation resulting in insufficient increase of MBF from rest to stress. Noninvasive diagnosis of microvascular dysfunction can be achieved by measurement of MPR and MBF on several imaging modalities, with PET having the most clinical and prognostic data thus far. CMR perfusion has been validated against invasive and non-invasive techniques, such as coronary angiography with FFR and PET for the assessment of microvascular dysfunction, and has shown robust accuracy [67]. Zorach et al. demonstrated that fully quantitative perfusion CMR can detect microvascular dysfunction in patients with angina and non-obstructive CAD as it shows reduced stress MBF and reduced MPR compared to normal control subjects [68] (Figure 2). Using invasive coronary angiography with FFR as a reference standard, Kotecha et al. demonstrated that in patients with regional perfusion defects, regional stress MBF > 1.94 mL/g/min matching coronary artery distribution is suggestive of regional microvascular dysfunction. Furthermore, in patients with no regional perfusion defects in keeping with a specific coronary artery territory, a global stress MBF < 2.25 mL/g/min is diagnostic of global microvascular dysfunction [12]. In a recent study by Rahman et al., transmural MPR and sub-endocardial MPR demonstrated excellent diagnostic accuracy in identifying coronary microvascular dysfunction in patients with angina and non-obstructive CAD. An MPR threshold of 2.19 yielded sensitivity and specificity values of 70% and 90% respectively while a sub-endocardial MPR threshold of 2.41 yielded a 95% sensitivity and 72% specificity [69]. As it is becoming clear that microvascular dysfunction is associated with less favorable outcomes, quantitative CMR is amongst the most promising tools in chest pain evaluation and risk stratification. However, more studies are necessary to assess its clinical utility and the impact of various treatment strategies in the improvement of MBF as well as patient prognosis. Moreover, inter-center reproducibility of the measurements needs to be performed before the application of the technique to daily clinical practice. Indications and contraindications of novel parametric mapping techniques in CCS are summarized in Table 1.

### 3.3. Prognostic Significance

Data regarding the prognostic benefit of quantitative perfusion mapping CMR are limited. However, a few recent studies have investigated the prognostic value of quantitative perfusion CMR analysis in patients with CAD. Sammut et al. demonstrated that ≥10% ischemic burden based on MPR was superior to visual assessment and provided incremental prognostic value over conventional risk factors, including age, sex, and LGE for the prediction of the composite endpoint of cardiovascular death, nonfatal myocardial infarction, aborted sudden cardiac death and revascularization after 90 days [70]. Knott et al. also showed that stress MBF and MPR were independent prognostic factors for death and major cardiovascular events [71]. Furthermore, a lower-than-normal MPR was associated with adverse clinical outcomes even in patients with no myocardial perfusion defects. In a recent study by Seraphim et al., both global stress MBF and MPR were independent predictors of all-cause mortality and major cardiovascular events in patients with prior coronary artery bypass grafting [72]. The above studies provide encouraging results on the value of quantitative stress CMR in clinical practice.

### 3.4. Strengths and Limitations

Automated quantitative perfusion CMR mapping has many advantages. Firstly, apart from its ability to identify the extent of ischemia in patients with multivessel CAD and differentiate from microvascular dysfunction, quantitative perfusion analysis can improve the differentiation of dark rim artifacts from true perfusion defects. Furthermore, the assessment of perfusion gradients (endocardial vs. epicardial MBF), provides further physiological insights for the evaluation of myocardial ischemia. Moreover, newer techniques allow for free breathing acquisition with motion correction, as well as pixel-wise quantification of MBF in a quick and accurate way. The software used for image reconstruction (Gadgetron framework) is widely available and may be used by all scan manufacturers, thus facilitating its clinical adoption.

On the other hand, significant variability in heart rate and breathing patterns may negatively affect the image quality. Furthermore, as with other mapping techniques, there is need for the establishment of normal ranges before routine clinical use. Lastly, the implementation of quantitative perfusion CMR has been limited so far to expert CMR centers, and it remains to be seen whether the technique can be incorporated into routine clinical practice.

## 4. Future Directions

T1 mapping and quantitative CMR using artificial intelligence and deep learning models have shown promise in the reliable non-invasive assessment of patients with chronic coronary syndromes. More specifically, deep learning techniques which allow preprocessing for quantitative CMR have already been developed and incorporated in pipeline processes, resulting in faster and accurate image acquisition and post-processing [73]. Measurement of myocardial perfusion pre- and post-invasive intervention could also be of significance in evaluating the results of revascularization. Application of artificial intelligence for image acquisition, analysis and data interpretation is a novel, highly promising field that will diminish the time and cost required for novel CMR techniques, improve accuracy and facilitate diagnosis and eventually patient care [74,75]. VNE has been successfully applied in patients with previous myocardial infarction [44] and has the potential to reduce scan times and costs, and improve the clinical accessibility of CMR in the near future. Although their role in the diagnosis has been established, prospective multicenter clinical trials are warranted to establish the added value of quantitative stress CMR and T1 mapping in clinical outcomes of patients with chronic coronary syndromes.

## 5. Conclusions

Quantitative stress CMR and T1 mapping are evolving non-invasive imaging techniques which hold promise for the assessment and management of clinical decisions in patients with CAD. Artificial intelligence, machine learning and automation in image acquisition and analysis are expected to increase the availability and clinical application of these techniques in daily clinical practice and to improve patient care.

## Figures and Tables

**Table 1:** Indications and contraindications of novel parametric mapping techniques in CCS.

	Indications	Contraindications
T1 mapping	Suspected or known CAD for differentiation of normal from ischemic and infarcted myocardium Chronic myocardial infarction: identification of scar; quantification of the extent of fibrotic tissue	Contraindications for vasodilator pharmacological stressors General contraindications to MRI Abnormal heart rate is a relative contraindication as it affects image quality
Quantitative myocardial perfusion mapping	Calculation of myocardial blood flow and myocardial perfusion reserve in patients with known or suspected CAD Detection of multivessel CAD Diagnosis of microvascular dysfunction	

## Data Availability

Primary data in the form of imaging data are available on request with restrictions for patient privacy.
